# Exploring the perspectives of healthcare professionals on providing supported asthma self-management for Bangladeshi and Pakistani people in the UK

**DOI:** 10.1371/journal.pone.0302357

**Published:** 2024-06-10

**Authors:** Salina Ahmed, Hilary Pinnock, Liz Steed

**Affiliations:** 1 The Institute of Population Health Sciences, Asthma UK Centre for Applied Research, Queen Mary, School of Medicine and Dentistry, University of London, London, United Kingdom; 2 University of Greenwich, School of Health Sciences, London, United Kingdom; 3 Usher Institute of Population Health Sciences and Informatics, Asthma UK Centre for Applied Research, School of Medicine, The University of Edinburgh, Edinburgh, United Kingdom; Bay Area Hospital, North Bend Medical Center, UNITED STATES

## Abstract

**Background:**

Self-management support improves asthma outcomes and is widely recommended in guidelines, yet it is poorly implemented in routine practice. There may be additional challenges in the context of ethnic minority groups, where making sense of culture may be necessary. This study aimed to explore the perspectives of healthcare professionals on supporting UK Bangladeshi and Pakistani patients to self-manage their asthma.

**Methods:**

One-to-one semi-structured interviews with professionals (primary and secondary care; medical and nursing) who routinely provide asthma care to Bangladeshi or Pakistani patients. Topics addressed included perceptions of professionals in supporting patients with asthma self-management and ideas for improving culturally competent care. Data were analysed thematically.

**Results:**

Nine professionals, from a range of ethnic backgrounds, with considerable experience of treating patients from these communities were interviewed. Despite organisational restrictions (language and time/resources) and expressed gaps in cultural knowledge and training, all interviewees reported attempting to tailor support according to culture. They used their perception of the patient’s culture (e.g., big families and family involvement), integrated with their perception of patients’ ability to self-manage (e.g., degree of responsibility taken for asthma), to formulate theories about how to culturally adapt their approach to supported self-management, e.g., supporting barriers in understanding asthma. There was consensus that gaps in cultural knowledge of professionals needed to be addressed through training or information. Interventions recommended for patients included basic education, group meetings, and culturally relevant action plans.

**Conclusion:**

In the absence of formal training and constrained by organisational limitations, self-management support was adapted based on personal and professional perception of culture. These ideas were based on experience and formulated a chain of reasoning. Professionals recognised the limitations of this approach and potential to overgeneralise their perceptions of culture and adaptations of supported self-management. Interventions were desired and need to address professional training in cultural competence and the provision of culturally relevant materials.

## Introduction

People with asthma are living with a variable condition [1–3]. Clinical guidelines widely recommend that healthcare professionals (HCPs) provide supported self-management which improves asthma outcomes. This includes providing patient centred education, personalised written action plans, supported by regular professional reviews [1–6]. However, South Asians in the UK tend to have poorer asthma outcomes compared to the indigenous White populations and other ethnic minority groups in developed countries, such as Canadian Chinese people [7–10]. To attain equitable outcomes, it has been argued that the mainstream healthcare system and individual HCPs need to become more culturally competent by adjusting and tailoring self-management support for individuals from different cultural backgrounds [7,11,12]. Accounting for factors such as differences in language, role of social support, acculturation (which is the cultural changes of a patient influenced by encountering another mainstream culture), and other perceived social and economic capital [12–14].

Cultural competence is the professional’s capability to be aware of, make sense of, and apply cultural knowledge, skills, and resources in cross-cultural situations to maintain good quality care [15]. At an organisational level, cultural competence are the behaviours, attitudes, and policies that influence the process of care and determine the workforce that carry out tasks within the system [16]. To try and improve cultural competence, a commonly implemented approach is ’ethnic matching’ of providers and patients, with the assumption that this will overcome language barriers and build bridges towards understanding a patient’s culture and health behaviour [17]. However, there is limited evidence on whether this approach is successful, including with South Asian populations with asthma, as it may be too simplistic and does not consider important issues, for instance differences in culture such as acculturation between patients and the HCPs [18,19]. The foundation of achieving cultural competence is deeply rooted in understanding what is meant by culture, however due to its dynamic nature it has no single or universal definition. We can understand culture as a subjective reality experienced by an individual or those who observe it, of which cultural knowledge, such as its practices, symbols, ideas, beliefs, norms, and values, can be learnt or transmitted socially and across generations [20,21], allowing meaningful definition of a society, including identity, relationships, and boundaries with others [22,23].

Berry’s Acculturation Model [14,24,25] suggests that individuals from ethnic minority groups adapt over generations to a mainstream environment in one of four ways: integration, assimilation, separation, and marginalisation (see Table 1 for full definitions). Reflecting these adaptations, there are four ways in which acculturation occurs in the mainstream group: multiculturism, melting pot, segregation, and exclusion (see Table 1). The ability to provide supported self-management to ethnic minority populations (South Asian groups in our study) is likely to be influenced by acculturation in both those from minority individuals/groups (patients and their cultural group), and the mainstream system (the HCPs being reflective of the mainstream system) [15]. Whilst some studies have explored how patients from ethnic minority groups self-manage their asthma [26–28], rarely has the perspectives of HCPs and their cultural competence to provide supported self-management been examined, or research undertaken on approaches that could develop cultural competence within healthcare organisations [18,19].

**Table 1:** Acculturation strategies between minority individuals/groups and the larger mainstream society [14,24,25].

	Minority individuals/group adaptation strategies within the mainstream	Mainstream adaptation strategies influencing the minority individual/group
1)	*Integration* Where individuals maintain their original culture and also integrate with the mainstream culture	*Multiculturalism (similar to integration)* When cultural diversity is fully supported as a feature of the mainstream society. The correct atmosphere needs to be in place for the integration strategy to be chosen and sustained
2)	*Assimilation* Where individuals disconnect from their original culture to fit in with the mainstream culture	*Melting pot (similar to assimilation)* Where the mainstream society is more dominant than the individual’s own cultural group
3)	*Separation* Where individuals hold on to their original culture and avoid any adaptation or contact with the mainstream culture	Segregation (similar to separation) Where the mainstream society tries to force separation of the individual from their society
4)	*Marginalisation* Where individuals lose maintenance and contact with both original and mainstream cultures	*Exclusion (similar to marginalisation)* When both mainstream society and the individuals cultural group force exclusion through various societal factors

In this study, we aimed to explore the perspectives of HCPs on providing care in the exemplar context of supporting Bangladeshi and Pakistani patients living in the UK to self-manage their asthma. A secondary aim was to explore interventions and the professional and practice development that HCPs believe would be needed to promote cultural competence in supporting asthma self-management for Bangladeshi and Pakistani patients. A separate study looking at the perspectives of the patient has been reported elsewhere [29].

## Materials and methods

### Study design and approvals

This qualitative study was conducted in 2017 with ethical approval from South Yorkshire Research Ethics Committee (IRAS ID: 200955); governance approval from the Health Research Authority (REC: 16/YH/0524), and sponsorship by Barts Health NHS Trust. All participants provided written informed consent.

### Participants

We included perspectives of HCPs on providing care for Bangladeshi and Pakistani patients as this study builds on our previous qualitative study interviewing patients in these cultural groups that explored self-management support for asthma [29]. This identified that despite similarities in patient experiences, sub-cultural diversity was important, such as the use of oral languages that have no written form, which specific to Bangladeshi patients. Professional groups of primary and secondary care clinicians identified as important by Bangladeshi and Pakistani participants were purposively sampled in this study [29]. We advertised the study via email to professionals who worked in three London Boroughs (City of London, Tower Hamlets, and Waltham Forest) with majority populations of Bangladeshi and/or Pakistani patients. We placed no restrictions on the clinical professional group or setting in which they worked. In this article, the word ‘Caucasian’ is used only if participants described their own ethnicity in this way, and if they used it during the interviews. We included three items from the Suinn-Lew Asian Self-Identity Scale (SL-ASIA) to estimate the degree of acculturation in the sample. Questions included: How do you identify yourself? What languages can you speak? How would you rate yourself (in relation to identification with culture)? To our knowledge, there are no existing scales validated for specifically measuring the acculturation of HCPs [30,31].

### Study recruitment

Doctors and nurses at primary care (GP practices) and secondary/tertiary care (asthma clinics and emergency medicine departments) were recruited. Eligible participants were provided with information about taking part in the study, face-to-face or via email, and they were invited to respond with an expression of interest form either using a prepaid envelope or by email. According to our sampling strategy, participants were then invited to a face-to-face interview with the researchers and provided written informed consent.

### Data collection

One-to-one audio-recorded semi-structured interviews were carried out by one researcher (SA; female; British South Asian PhD student) with previous qualitative research experience, until data saturation was achieved with respect to the research questions (i.e. when no new perspectives were emerging) [32]. One interview was conducted by another researcher (SM; female; White British Irish PhD student), to reduce interviewer/interviewee bias resulting from the primary researcher (SA) being known to one HCP. The interview schedule consisted of six main topic areas and several prompts designed to explore perceptions of supporting Bangladeshi/Pakistani patients with their asthma self-management and to elicit any ideas for improving culturally competent care (see Table 2). Interviews were carried out at the HCP’s place of work (GP surgeries; hospitals) or at the university and lasted 20 to 90 minutes. Reflexive notes in a research journal were taken by the researchers after each interview and emerging findings were discussed with the study team. The COREQ checklist for reporting qualitative research was used to ensure comprehensive reporting of methodology and analysis and enhance credibility in the findings.

**Table 2:** Interview schedule.

Questions and prompts
1. Tell me about your profession and experience of working with Bangladeshi and/or Pakistani patients? 2. Tell me about your views on how Bangladeshi/Pakistani patients manage their asthma? 3. Tell me about how you use asthma guidelines to support Bangladeshi/Pakistani patients with their self-management? 4. Tell me about what your relationship is like with Bangladeshi/Pakistani patients? 5. Which other professionals do you work with in partnership to provide asthma self-management support to these patients? 6. Tell me about any ideas or suggestions you may have on how to better support these patients with their asthma self-management?

### Data analysis and interpretation

The interviews were digitally recorded and transcribed. QDA Minor (version 2.0.1) was used to support data analysis. The data were analysed using an inductive data driven approach based on thematic analysis methods suggested by Braun and Clarke [33]. Themes were identified at a latent level (surface descriptions and underlying ideologies, assumptions, and concepts that shape these descriptions), and using contextualist framework (individual and sociocultural influences on meanings) [33]. All interviews were independently coded by one researcher (SA). Two further researchers (LS, HP) independently coded six transcripts. Coding was then compared to ensure reliability of coding (SA, LS, HP, JP). Any discrepancies were resolved by discussions between reviewers until consensus was achieved.

## Results

### Participant characteristics

The characteristics of the nine HCPs interviewed are shown in Table 3. All recruited participants completed the interview. They represent general practitioners, asthma specialist physicians, a doctor from an emergency medicine service, and two nurses with community/primary/secondary care expertise. There were five females and four males, between the ages of 34 to 58, from a range of ethnicities, self-identifying as: Bangladeshi, Irish, Korean, Spanish, and White Caucasian. All HCPs reported considerable experience of working with the Bangladeshi and/or Pakistani patients (ranging from over five to thirteen years). The degree of acculturation in the sample revealed that [31]:

- Four HCPs identified themselves as British, and five HCPs identified themselves as British alongside their ethnicity
- All HCPs identified themselves as westernised
- Four HCPs spoke another language alongside English, such as Standard Bengali or Bengali Chittagong dialect, Korean, and Spanish. Five HCPs could only speak in English

**Table 3:** Participant characteristics.

Profession	Ethnicity	Gender	Selected SL-ASIA questions		
			How do you identify yourself?	What language/s can you speak?	How would you rate yourself?
GP doctor	Irish	Male	British Irish	Only English	Other: very westernised & Irish
GP doctor	Spanish	Female	British Spanish	Bilingual (English & Spanish)	Mostly westernised
GP doctor	Bangladeshi	Female	British Bangladeshi	Bilingual (Standard Bengali)	Mostly westernised
Asthma consultant	White Caucasian	Male	British	Only English	Very westernised
Asthma consultant	Bangladeshi	Female	British Bangladeshi	Mostly English, some Chittagong	Very westernised
Asthma consultant	White Caucasian	Male	British	Only English	Very westernised
Doctor (emergency medicine)	Korean	Male	British	Mostly English, some Korean	Other: mostly westernised & Korean
Nurse specialist	Irish	Female	British White Caucasian	Only English	Other: mostly westernised & Irish
Primary care nurse	White Caucasian	Female	British	Only English	Other: mostly westernised & Irish

### Overview of findings

**Fig 1:**
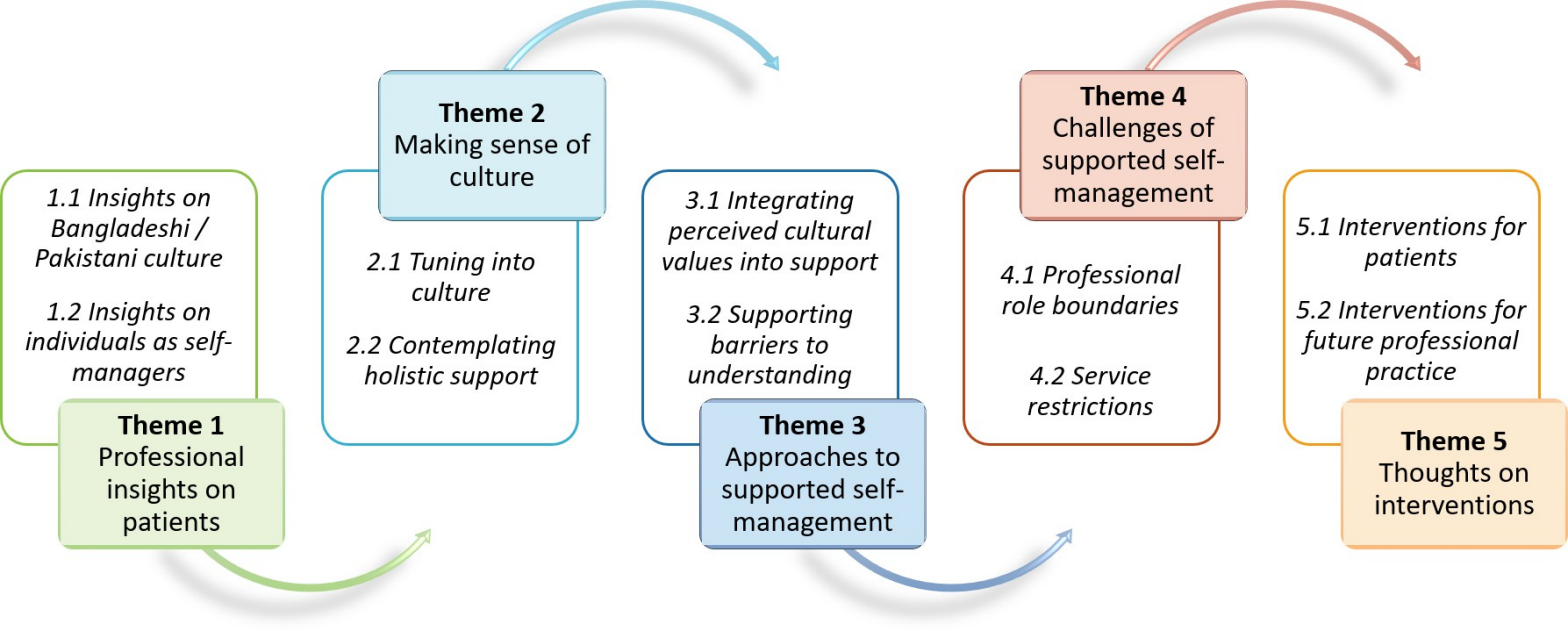
Thematic schema: Chain of reasoning behind providing supported self-management to Bangladeshi and Pakistani individuals living with asthma.

Fig 1 illustrates the five interrelated themes associated with providing supported self-management to Bangladeshi and Pakistani patients, and how these formed a chain of reasoning. Findings are presented under these five themes, each with two further subthemes. S1 Table provides some additional illustrative quotes.

#### Theme 1: Professional insights on patients

Theme one illustrates the perspectives of HCPs on Bangladeshi and Pakistani culture and its influence on asthma self-management behaviour.

*Subtheme 1.1. Insights on Bangladeshi/Pakistani culture.* HCPs generally described Bangladeshi and Pakistani culture as fixed and unchangeable, although there was a recognition of different migratory generations, such as first, second, third generations, and the influence this had on culture. Generational influences were typically judged by age; older patients were perceived as more likely to be first generation migrants than younger migrants. Speaking English was another characteristic equated with a particular set of cultural beliefs about patients and their self-management behaviours. Language was a major barrier to communication, even for professionals with similar ethnicity to patients who were not bilingual, and many HCPs commented that supporting self-management was problematic because of communication difficulties with older or first-generation patients. In contrast, patients who could speak English, which was perceived to be a western asset, were believed to be easier to support or treat with the assumption that there were fewer cultural differences between patients and professionals.

“*I think that the*, *the two Asian groups of people in this cultural group we are talking about*, *and that ties into those who speak English well and those who do not speak English well*. *So*, *for the people who speak English well*, *I think that they are quite capable of using the current information*, *guidelines*, *action plans*, *and everything*. *For people who do not speak English very well*… *There is likelihood that they may have also*, *different*, *alternative beliefs as well*, *that they would be more likely to have beliefs in other treatment systems etc*.*” (GP*, *participant 1)*

Older age of patients was perceived to be related to resistance to changing attitudes to self-management due to having fixed traditional ideas about asthma and its treatment. On the other hand, resistance to self-management advice was also attributed to a few younger English-speaking female patients, who disowned their asthma to avoid being socially blamed for adopting culturally frowned upon or harmful behaviours for women, such as smoking or misusing substances as a weight control strategy.

“*Then you try to give smoking advice*: *‘reduce smoking’*, *‘stop smoking’… But they’re like*, *’Nah I*, *I just don’t have asthma*, *I don’t have it’*. *So*, *they’re complete barrier*, *so they can understand English*, *and they can speak it*, *and you can do a self-management plan*. *They’ll take it home*. *They’ll come back in a couple of weeks’ time and say*, *’I’ve stopped using it’… ’Did you stop smoking*?*’… ’No*, *that has no impact on my lifestyle and my health’*, *so you’re*, *on a full cycle again” (Nurse specialist*, *participant 7)*

Most HCPs felt under-educated in culture and its influence on health, and therefore perceived that ethnic matching of patients with staff (considered as insiders to the group), may be helpful to provide insights into cultural problems and motivate patients, including primary care receptionists. In contrast to other cultures, there was a general consensus that big families were an important part of Bangladeshi/Pakistani culture, and their involvement was helpful in most cases and implied support, particularly for providing language translation.

“*What tends to be better about a lot of patients from Indian subcontinent is that they usually come with family*. *More than Caucasian who don’t*. *They come alone*, *usually*. *And so*, *you know*, *especially sort of old*, *older people nearly always come with the younger person from the family*, *and partly that is for*, *to translate*. *But at least you’ve got somebody there from family*, *who is*, *helping with that*. *And they tend to have more family support as well*, *so in a way*, *you know compliance should be better*, *shouldn’t it*? *Because they’ve actually got family around who can help them with*, *with inhalers and things like that and taking everything regularly” (Consultant*, *participant 6)*

There was also frustration that some first-generation patients were strongly influenced by those around them, being guided by advice, gossip, and reassurance.

“*There are many patients who are good with their asthma*, *so I think there’s a lot of discussion between*, *rather than just going away and taking your medication*, *they may well go back*, *and then there’s a lot of discussion around what they should do based on*, *what other people tell them*. *There’s a lot of gossiping*, *and that may be their main influence*, *rather than the professional” (Consultant*, *participant 5)*

A few HCPs perceived religion to be a part of culture whilst recognising that people differ in their extent of practice, and that it is a positive influence on self-management, as religious teachings encouraged patients to be responsible for their health.

“*I think with all religions there’s*, *there are a lot of people signed up*, *and the question is how deeply the beliefs come through*. *I would think that most religions*, *would put a responsibility on individuals to look after themselves*, *and if*, *whatever religion you take*, *if people were to follow it properly then that would be a priority in their life” (GP*, *participant 1)*

There was a broad recognition that patients, and their family members, tended to have a lot of ‘anxieties’ (as described by HCPs), and other emotional issues such as depression and anger that can worsen asthma symptoms, especially in the first generation. This was believed to be related to a cultural problem, along with the time to air such concerns in a restricted consultation, and limited access to psychological services.

“*The understanding around the treatment—there was often a cultural problem because*, *they didn’t*, *the people who were the more difficult ones to treat*, *didn’t have Western knowledge of treatment*, *and were very anxious generally*, *and had so many anxieties that it was difficult to give them instructions because they often talk so much about their anxieties that there wasn’t enough time in the consultation to make you feel convinced they really understood what you were asking them to do” (Consultant*, *participant 5)*

*Subtheme 1.2. Insights on individuals as self-managers.* HCPs described Bangladeshi/Pakistani patients as not engaging properly with their health by taking little responsibility for controlling their health and considered that they were too dependent, especially the first generation, on family support, who may be children or grandchildren looking after their elders, and professional advice.

“*Sometimes they do what you tell them*, *and sometimes they expect that advice*, *to follow that advice very carefully—maybe without trying to understand themselves*, *self-manage themselves*, *so that part of responsibility in their own treatment may be is missing a bit in that community*, *I would say*. *I’m not sure if it’s wrong but instead of maybe a bit more ‘proactive’ way of understanding your asthma*. *So maybe that’s the*, *maybe in terms of how to manage your own asthma*, *because it’s very much*, *‘you have to decide your own management depending on your symptoms*, *your triggers’*, *so maybe that is missing a bit…” (GP*, *participant 2)*

Over-reliance on the reliever inhaler as a short-term management strategy was described as a behaviour common to this culture, often reinforced by family, and related to the beliefs of the acute nature and short-term treatment solutions to asthma. Patients were perceived to have little understanding of asthma, self-management, and the consequences of preventative asthma treatment. This was generalised and described as a cultural problem, particularly for Bangladeshi patient, although the diagnosis of asthma was believed to be commonly known in the community.

HCPs were aware of the unsubstantiated assumptions they were making on their insights on patients as self-managers, such as providing advice on medication during Ramadan, and they were concerned that they were not always able to give clear explanations. They also recognised patient concerns about medication, for instance its side-effects such as addiction and depression, its social concerns such as weight gain and embarrassment of using inhalers in front of others, and the lack of religiously compliant medications such as ethanol-based inhaler preparations.

“*I used to find a lot of problems with inhaler technique*. *The issue*, *if there were issues*. *It was things like difficulty around using the actual device*. *They just wouldn’t get it*. *A lot of them*, *and I don’t know whether that’s there wasn’t enough time*, *translating how to use it or whether the concept of using inhaler would never quite sink in*, *where it was used more like a breath freshener*, *and also*, *I suppose*, *that was one—the actual physical taking” (Consultant*, *participant 5)*

Generally, patients were perceived to have positive attitudes towards HCPs, although some reported that Bangladeshi patients tended to trust doctors more than nurses. Doctors believed that nurses should be valued more because of their role in supporting self-management. Conversely, nurses noted that as patients did not perceive them as a medical professional, patients were able to fully express themselves.

“I *think some Bangladeshis*, *and this is again stereotyping them*, *used to not value a nurse*, *and to me I really think it’s so important that we ‘big up’ the nurses*, *because the nurses know more about inhalers*. *They have more time*, *and they understand a lot more about asthma than probably I do*. *But yet*, *they [patients] would come to me and say*, *’Oh doctor*, *what should I do*?*’ And I’d be*, *’Why have you come to me and not the nurse’ (laughs)*. *So*, *I think that’s the only thing is that they need to understand the value of the nurses and the pharmacist*, *to help*, *and engage*, *and empower them” (GP*, *participant 3)*

Some HCPs were frustrated with patients who travelled to South Asia and came back with contradicting medical opinions on self-management.

“…*When they travel*, *and they would travel to their home country and get the—get some help from the medical people over there*, *and sometimes it wouldn’t be necessarily the management that we would’ve*, *advised*. *So*, *they would usually come and say*, *“My doctor in Bangladesh*, *for example*, *have told me to do this and that*, *or they have advised me to take another chest x-ray or get a CT scan and so on”*. *So*, *there are very varied messages that they receive I think*, *yeah” (Emergency medicine doctor*, *participant 9)*

#### Theme 2: Making sense of culture

Theme two illustrates the process of reasoning, contemplating, and validating participant’s professional insights on Bangladeshi/Pakistani culture and considering how to adapt support.

*Subtheme 2.1. Tuning into culture*. HCPs reflected on their personal/professional identity and experience of Bangladeshi/Pakistani culture and tried to make sense of patients’ self-management behaviour. HCPs used their experience to reflect on the ideal culturally sensitive supported self-management strategy. Assumptions were based on repeated contact with, and what they had read about, subcultural groups they most encountered. For example, experience with Bangladeshi patients and the Bengali/Sylheti language shaped understanding of the challenges of literacy:

“Yeah, *I’m unsure at the moment*, *how we can make it [referring to action plans] adaptive to the Bengali speaking patients and I would not say doing it in their own language is the right way*. *Because I think a lot of literature*, *says it’s not always helpful to translate into Bengali*, *and that’s because the amount of money costs*, *time it takes to translate*, *then the back translation is not always perfect*, *and then people who can actually read Bengali often can read English*, *and also*, *if they can’t their family members can*, *so…” (GP*, *participant 3)*

Instead of any formal education, all interviewees relied on generating theories based on speculations that centred on their personal/professional experience to make sense of a patient’s self-management behaviour, using words such as ‘I’m guessing’. For instance, why patients were very anxious:

“*Yeah*, *the sort of ones who can’t speak English*, *who are very anxious*, *and it will just be really useful to know what is it that would make them less anxious*. *And make them maybe more*, *you know*, *adherent to their treatment*, *because I’m*, *I’ve come up with all these theories*, *but I don’t actually know” (GP*, *participant 2)*

*SUBTHEME 2.2. Contemplating holistic support.* HCPs contemplated the support for Bangladeshi/Pakistani patients within the broader concept of supporting all patients holistically. Treating patients holistically meant addressing physical and mental co-morbidities, not restricted to asthma alone, in supported self-management:

“*And you might say how can we make this piece of paper more useful to Bangladeshi patients*. *I think this is part of a broader picture that we are looking in general practice*, *which is we are being asked to do plans for everyone*, *you know*: *COPD*, *diabetes*, *frailty*, *cancer*, *blood pressure—and asthma just happens to be one of them” (GP*, *participant 3)*

The HCPs were cognisant of the balance between treating all patients the same regardless of ethnicity, by typically making comparisons to White British people with asthma, but they were also conscious of being responsive to culture, for instance narratives around language involved comparisons to other communities such as European and Turkish communities:

“*…Maybe with children and mums*, *that there are*, *is a little bit of reluctance of using inhalers*. *If the child has asthma and they’re worried if they keep using a brown inhaler*, *if they gonna get addicted to it*. *But*, *say*, *that’s possibly not any more different to one of my White Caucasian families” (GP*, *participant 3)*

#### Theme 3: Approaches to supported self-management

HCPs suggested supported self-management strategies for Bangladeshis/Pakistanis based on their professional insights and how they made sense of culture.

*Subtheme 3.1. Integrating perceived cultural values into support*. All the HCPs, including professionals from the Bangladeshi/Pakistani ethnicity, believed it was helpful to involve the family in support. Family acted as primary translators, and HCPs often pressurised patients to bring family into consultations for this purpose, noting that family have an obligation or a responsibility for this. In addition, family were perceived to be helpful in remember and understand information and providing on-going support for patients. This attitude assumed consent to disclose a diagnosis to supportive family members. The issue of obtaining consent before discussions was only touched on by a nurse specialist who was aware of conflicts for (often younger) patients, who valued privacy due to smoking, drug use, and perceived stigma of asthma hindering marriage prospects. Most HCPs also pointed out limitations of involving family support, for example it can exclude the patient from the conversation or prevent disclosure of sensitive personal information, inadequate translations can lead to misinformation, and patients may abrogate responsibility for their health to others.

“*Sometimes I mean there’s no problem having more people in the room*, *that’s fine*. *Sometimes*, *people*, *family members are dragged into the room who are not interested*. *They’re sitting in the back*, *doing other things*. *That’s very distracting for everyone*. *Sometimes the actual patient is very much excluded from the conversation unfairly*, *that the family don’t seem to want to involve them*, *and actually want to talk over them*, *and that I think is very problematic” (Consultant*, *participant 4)*

Most secondary care HCPs recognised the need for emotional support, especially for anxiety, but only a few accommodated this (including primary care professionals) by signposting other services. One asthma clinic employed physiotherapists to manage hyperventilation, and another utilised external psychologist services. Some highlighted cultural barriers, such as stigma, in accessing psychological services. Most HCPs believed that they and their colleagues needed to be more aware of medicine adherence, as well as other religious self-management strategies during Ramadan, so they could confidently provide advice around balancing fasting, health and safety, and medication. Unlike with other conditions, such as diabetes, advice on asthma during Ramadan was described as ‘contradictory’, with limited guidance for professionals. In the absence of knowledge, most HCPs guessed what religious advice would consist of, and often referred patients to the Imam.

“*…Only during Ramadan*, *where I think there’s a debate as to whether they take their inhalers or not*, *and I think whoever’s an opportunist could say*, *‘I’ve seen that GPs find it helpful to give some guidelines on*, *during Ramadan*, *what you like to do with your inhalers and your medication’*. *Because it matters in diabetes but there’s nothing*, *not*, *I’ve seen on asthma” (GP*, *participant 3)*

A few HCPs thought that there was no need to treat patients differently during Ramadan since the preventer, used either once or twice a day, could be adjusted into hours when patients do not fast, and the reliever should not be necessary. Some HCPs suggested balancing religious beliefs and medical safety by placing alerts on the prescribing systems to warn HCPs that an inhaler contained alcohol, or that a flu vaccine used gelatine. All HCPs were accepting of complementary and alternative medication (CAM), if use was ‘complementary’ to biomedical treatments, particularly if it made patients feel better. In contrast, if use was ‘alternative’ to medication, it was described as harmful and costly, with examples of patients holding erroneous beliefs that natural remedies were always safe. Most HCPs felt ill-prepared to discuss CAM with Bangladeshi/Pakistani patients, although a few described discussions with White British patients.

*Subtheme 3.2. Supporting barriers to understandin*. Different forms of education were provided by professionals, including advice, scenarios of situations, teaching sessions, posters, leaflets, and group activities. To overcome language barriers in understanding, a few HCPs provided audio-visual education which focused on visual demonstrations or lung models:

“*I show them diagrams*, *so they can’t actually go wrong*, *which is from*, *it’s not actually Asthma UK*, *it’s called Right Breathe*, *and it’s brilliant because it comes up with the asthma inhaler they’re on*, *and then it has a video of how to do it*. *There is no way someone cannot understand somebody watching the video because there is no language involved*, *it’s just*, *basic*… *Like I say*, *there may be a language barrier there*, *but if there’s nobody with them*, *they’ve got the video” (Primary care nurse*, *participant 8)*

Some HCPs highlighted the need for follow-up consultations to check patients’ understanding of education and whether they implemented learning, recognising the potential barriers to understanding in these communities. Most HCPs wanted to provide more than what was routinely offered, such as repeated online audio-visual education. Overwhelming patients with information was a concern and some HCPs believed that ‘going back to basics’, and repeating information to reinforce understanding was important. Most HCPs provided reassurance and tried to normalise the process of taking inhaled steroids daily.

“*Just repeating it a lot until hopefully patients understand and follow the plan” (Consultant*, *participant 4)*

Most HCPs were empathic towards cultural and religious beliefs, attitudes, and values, as this allowed them to understand the patient’s self-management strategies. All HCPs reported that a trusting relationship with patients and their family over time helped break down barriers, reduce anxiety, and improve understanding.

“*Because they look at me and think*, *‘You don’t understand our values*, *so I’m not going to listen to you’*. *So*, *if you chip it back and get an understanding*, *‘I know what you’re thinking*, *and I know how to explore it’*. *Because you need to understand that they*, *the family*, *would have to support them*. *How do you get into the family’s mind-set*? *How do you get into mum and dad’s mind-set*? *How do you get into brothers and sisters’ mind-set*? *What are their values*? *Why do they not want to use it*? *Get a better understanding of the person*. *Don’t see them as a patient*. *See them as a person*, *because there’s a lot more going on in their head*, *than we know” (Nurse specialist*, *participant 7)*

A few HCPs described ethnic matching of professionals to patients as an ideal way to develop rapport with patients.

#### Theme 4: Challenges of supported self-management

Theme four illustrates the organisational challenges in the healthcare system which influence the practical ability to implement culturally competent supported self-management.

*Subtheme 4.1. Professional role boundaries*. HCPs recognised subcultural differences and that their perceptions and understanding of Bangladeshi/Pakistani patients were confined to the population with whom they had the most contact with. Most participants had more experience with Bangladeshi patients. Time was limited, and doctors prioritised their role to diagnose and offer specialist advice on medicine and prescribing and suggested that other team members should manage other aspects of treatment; nurses were often mentioned as valuable for supporting self-management. Although, nurses reported time as a barrier, sometimes they became very involved with patients and occasionally performed tasks beyond their role, for example arranging repeat prescriptions and appointments in primary care.

*Subtheme 4.2. Service restrictions*. The organisation of the healthcare service created barriers, such as poor communication between primary and secondary care. Secondary/tertiary care HCPs wanted better access and communication with GPs to improve ongoing supported self-management, particularly after discharge and during follow-ups. Conversely, a primary care nurse sometimes disagreed with asthma consultants, for example on the choice of inhaler device, but felt they had to comply because consultants were the ‘experts’. Previous examples of cross-sectoral working were cited, including holding joint committees and update or education meetings. Most HCPs thought that there were gaps in the services for dealing with language barriers. The use of interpreters, health advocates, translated written information, and Language Line (a three-way telephone interpretation service), was limited due to access/booking issues, time/delays, and cost.

“*When you see them come into hospital and you think ‘how does a doctor do an assessment when they don’t even have an interpreter*?*’ ‘How do you get to understand*, *what is*, *what they’re feeling’*. *So*, *even the elderly*, *come in and you think*, *you’ve just done an assessment*, *but how have you done a physical assessment*? *I’ve just stood and looked at you*, *the patient has gone*, *nodding her head but didn’t understand*. *Is that agreement*? *Are they saying*, *’Oh no*, *I don’t have*…*’*, *so it’s very much*, *they need*, *I think they should be utilising interpreters a lot more” (Nurse specialist*, *participant 7)*

One GP surgery described a good and consistent presence of health advocates incorporated into their stop smoking and teaching session services for most languages. It was suggested that guidelines should make recommendations for reducing language barriers and signposting sources of help. Most HCPs agreed that action plans were important but difficult to implement, specifically adapting them culturally. One GP practice described implementing action plans as ‘on their wish list’, unprioritised due to time and service constraints. Even ignoring oral languages and back translation issues, available templates were perceived to be complicated, having too much or irrelevant information, and conversely, not enough information for the range of people who need it. Some services, such as emergency departments, did not consider providing a plan was within their remit.

“*I think if you do a plan as a doctor or a nurse*, *it’s only as good as a piece of paper*. *What you need to do*, *is make sure that the school adopts it*, *that the child adopts it*, *that the family adopts it*, *that if you say*, *’Does everyone know where the inhalers are kept*? *Do they have their own place*?*’ So*, *all of that needs to go into the management plan*, *and you might say*, *‘how can we make this piece of paper more useful*, *to Bangladeshi patients*?*’” (GP*, *participant 3)*

#### Theme 5: Thoughts on interventions

Theme five illustrates HCP’s thoughts on potential interventions for patients and for their future professional practice (see Table 4 for more details). Interventions recommended for patients were often to overcome language difficulties rather than for those who could speak English, and included basic education (e.g., workshops, audio-visual education, and leaflets on Ramadan), group meetings (e.g., self-help groups and social prescribing), and culturally relevant action plans. All HCPs suggested that cultural training would be beneficial for their professional development, since in the absence of available training they had to rely on personal experiences, for example religious teachings about asthma, cultural beliefs, communication strategies, and explanations for self-management behaviour. Patient involvement in training was suggested by some HCPs, such as communicating through storytelling (described as a significant cultural value) and learning through empathy. There were some concerns about potential training, for example time, length of training, resources/funding for training to be developed and delivered, and content. A focus on asthma self-management in South Asians may be too specific for doctors who deal with broader health issues. Some HCPs indicated that population-specific information and research would be helpful.

**Table 4:** Suggested service development interventions for patients, professionals, and practice/organisations.

*Interventions for patients*	
Basic education (such as workshops, open days, audio-visual education, and leaflets on Ramadan) that could be delivered in settings such as primary care and mosques:	*“So*, *it’s ongoing education that can start locally*, *and where better than your mosque isn’t it*? *They’re not going to turn up to community centre because sometimes the woman does not like to mix with the men*, *so you’re gonna think*, *how am I going to get sections in*, *and then community centres probably wouldn’t be the best idea*, *so you’d go locally to where the individual goes” (Nurse specialist*, *participant 7)*
Culturally tailored action plans addressing religion and language and incorporating the following ideas: non-textual, verbal, pictorial, simple, colourful, stickers and cultural icons and delivered on audio-visual formats e.g., DVDs, YouTube, and Bengali TV	*“It’s remarkably difficult*, *because I think they tried to plug it in TB*, *by translating it into Bengali and found a lot of people couldn’t read*, *so therefore actually it needs to be a verbal or*, *non-textual*, *asthma action plan*, *and that we haven’t yet got round to producing” (Consultant*, *participant 4)*
Group meetings that would foster confidence in self-help groups in the community or social prescribing that involved befriending	*“I think there’s a big place for*, *for multi-cultural self-help groups*. *To start with*, *to give people confidence*, *in managing it I think*, *people identify with people close with themselves*, *first of all*, *and they accept that advice initially”* (GP, participant 1)
*Interventions for future professional practice*	
Cultural training/information: cultural training to address gaps in cultural knowledge e.g., correct Islamic teachings on asthma, use of culturally appropriate terminologies, current explanations of self-management behaviours in the community (format: face-to-face, delivery: an HCP with asthma expertise and from a South Asian background with cultural knowledge). Some HCPs believed that patients should be involved in the training to experience cultural beliefs and attitudes and since it complements some current supported self-management strategies	*“There is no specific training anywhere within the NHS*, *I’ve never heard of*, *about cultural*, *specific factors for any of our patients*, *occasionally you get a word of wisdom from a consultant*, *whose training you about good phrases to use but*.* *.* *. *That would probably be in total across my twelve years of training about fifteen minutes of teaching moment” (Consultant*, *participant 4)*
Updated evidence on asthma outcomes amongst South Asians e.g., epidemiological data on asthma prevalence and unscheduled care	*“A bit more research out there detailing*, *you know*, *what the issues are*, *among- amongst that community and how much severe asthma there is out there amongst that population” (Consultant*, *participant 6)*
Understanding that patients’ explanations often come through telling their stories of living with asthma (stories were perceived to be a cultural value) and appreciating what this means for communicating with this population	*“I think people understand stories they like it*, *if you give positive stories you know*, *in Bengali*, *it’s ‘golphor’ [stories] you know*, *hearing the story about how positive stories came out of someone who*, *didn’t think that they would benefit from an inhaler and then actually started taking their inhaler*, *and then they took their prevention and then they had less hospital admissions” (GP*, *participant 3)*
Learning about the perceptions of Bangladeshi/Pakistani patients (e.g., from previous qualitative research, 26), especially since language issues translated interviews would help build insights on thinking styles	“*I would just be interested in a paper from you*, *with what sort of comments from the patients about*? *What is it that they feel they need*, *on asthma*, *and are not getting at the moment*?*” (GP*, *participant 3)*
Raising awareness: interview questions helped most HCPs realise and created an awareness of the way they see and provide supported self-management to their patients	*“Now that you ask me all these questions I can think about different things*. *So*, *then maybe once you know about all that*, *if you are with a Bangladeshi family*, *you can actually*, *start asking about those things” (GP*, *participant 2)*

## Discussion

### Main findings

We explored the perspectives of medical and nursing HCPs from across primary and secondary/tertiary care on providing supported self-management to Bangladeshi and Pakistani individuals living with asthma. HCPs were all aware of the importance of culture and tried to make holistic adaptations, within organisational constraints, but they also had to rely on their own hypotheses and experiences rather than any formal training. Professional perspectives of the Bangladeshi/Pakistani culture focused on the importance of family involvement, high ‘anxiety’ with reluctant of accepting psychological support, explanations through storytelling, patients’ having little understanding of and a passive role within self-management. Culture was often perceived to be static; a stronger influence on poor self-management when a person did not speak English, which tended to relate to age; and generation was defined around age. Acculturation was also reflected as synonymous to language (fluency in English) for younger patients who were also perceived to be better self-managers.

### Interpretation of findings

#### Perspectives on culture

There was a perception that Bangladeshi/Pakistani culture consisted of big families, involvement of whom could be either helpful, such as for language translation and support, or unhelpful, such as when family beliefs contradicted professional advice. Such beliefs risk overgeneralisation and stereotyping patients and could create undue pressure and stress if all patients felt obliged to involve family members in their self-management, for example when young patients are not aligned with these traditional cultural expectations due to acculturation. It is important to balance patient choice and the need for translation or extra family support [14,34]. As well as accounting for the volatility of social support over time, such as breakdown of relationships, and that support may be restricted to practical and moral assistance during symptomatic phases, all of which have been found in prior research [12,13,29]. Similarly, HCPs perceived that most Bangladeshis/Pakistanis did not have a good understanding of asthma medications, and Bangladeshis had trouble understanding asthma monitoring devices, such as peak flow meter, and this was described as a cultural problem. This is in line with prior research [27,35,36], including systematic reviews [7,37], that found South Asians have difficulty in understanding information around medications. Although, another study [29] revealed that Bangladeshis and Pakistanis knew more about their medication than about asthma itself, however some Bangladeshis found that asthma monitoring devices were difficult to understand and use, suggesting that perhaps understanding of asthma medications may be changing.

#### Perspectives on language

Nurses described alternative means of addressing language barriers by using audio-visual educational strategies, which were language free, to improve understanding of asthma medications, peak flow monitoring, and spirometry. An intervention mainly important for first-generation patients who had little English-speaking abilities. Although a Cochrane review [38], however, has shown that solely relying on language modifications is not enough to improve asthma outcomes. A previous systematic review [7] also found that audio-visual educational strategies improve inhaler technique in South Asians. Studies [26,39,40] have found that media materials and resources that present difficult information in the appropriate languages and dialects can help patients with low literacy despite ethnic background, such as illustrating asthma symptoms such as different sounds of cough (croup or whooping cough). Such interventions are extremely helpful for patients who conform to the ‘separation’ style of acculturation in speaking oral languages with no written form, such as Sylheti, and this needs further implementation and evaluation [26,39]. Prior research [41,42] showed that limited translation services are globally pertinent to ethnic minority groups, and lead to differential seeking of support from HCPs. A qualitative study [26] found that South Asian parents only sought help from HCPs who could speak the same language. Arguably, poor provision for language in organisations reflect institutional racism, since the needs of ethnic minority patients are not met and this is not directly obvious to users, although on a practical level it may also simply reflect an overburdened healthcare service and cost constraints [43].

#### Perspectives on cultural training needs

There is evidence to suggest aligned acculturation may be important in cultural competence. All professionals wanted cultural training to develop their cultural competence. They recognised that they were not experts in culture and some reflected that they were ‘extrapolating’ based on theories. This included a British Bangladeshi consultant and GP, who were both unable to speak Bengali reflecting the acculturation positions ‘assimilation’ (as an individual) or the ‘melting pot’ (as part of the mainstream healthcare service) [see Tables 1 and 3] [14]. This challenges the idea and studies [44,45] that ‘ethnic-matching’ (matching the ethnic background of HCPs and patients) is a sufficient intervention strategy. It opposes the common assumption that simply sharing the same ethnic background qualifies HCPs (or other staff) to be insiders of cultural knowledge, and hence that they might relate to or provide better care. This has the potential to reduce the ethnicity of HCPs (and their cultural competence) to a simple methodological approach of matching professionals with patients without prioritising the skills or qualities that an individual HCP brings to their profession [14,44]. Prior research [35,46] has shown that ethnic-matching overlooks the variety of South Asian languages and dialects spoken by professionals and patients from within the same ethnicity. The notion itself employs the assumption that there is harmony within cultures with natural/innate understanding and abilities, in addition to implying that all cultures need ethnic-matching [44,47].

This key issue pivots around how culture is defined, understood, and recognised, which needs to be ironed out in training, such as categorising language barriers and age/generation with poor self-management, as found in this study and others [48–50]. Previous qualitative studies [22,23,51] have suggested that professionals need adequate training, but how this should be delivered has been largely underexplored, as well as the organisational support that enable cultural competence. Other examples of gaps in cultural knowledge in this study include the use of theorisations and inaccurate terms, such as referring to patients as ‘Southeast Asians’ and using the word ‘Caucasian’ to describe White people. Training is needed to address the risk of stereotyping and over-generalisations, as with the professional approach to involving family in consultations and beliefs around cultural problems in understanding medications. Agreeing with previous research [52,53] this study found that interventions need to provide advice around fasting in Ramadan to avoid ‘guesses’ or referrals to ‘the Imam’ as someone all patients would want or have access to. Although, there is some guidance on self-management for other chronic illnesses during Ramadan, for example diabetes and cardiovascular disease, there is no similar advice for asthma [52,53]. Gaps in knowledge may mean religious teachings are extrapolated from one illness and applied to another inappropriately [52,54]. In addition, this study and prior research [51,52] have found that evidence and training on safety and efficiency of CAM would help HCPs to better advice patients on making informed decisions about herbal medicine, as well as allowing patients to openly communicate with HCPs about CAM use.

Although, some HCPs proposed that patients need to be seen as an individual, lending itself to cultural adaptations through holistic care, interventions need to target how supported self-management is understood and implemented as a responsibility for all professionals. Agreeing with prior research [13,29,55,56], doctors in this study believed that their role was based on disease management for asthma. This runs the risk that culturally competent care may not be given thoroughly by all professionals, until holistic individualised care is seen as the remit of all professionals.

### Strengths and limitations

A strength of this study was situating a complex understanding of the perspectives of HCPs (who had varying beliefs and views), with the recognition and focus on addressing cultural issues. Diverse professional groups, previously identified as important to this population were interviewed [29], but although we purposively sampled representatives of different professional groups working with either/both Pakistani and Bangladeshi patients, doctors out-numbered nurse interviewees, and most professionals in this study had more contact with Bangladeshi than Pakistani patients with asthma. We reached data saturation in terms of not hearing any new views in the latter interviews, but there remains concern that we may not have detected all possible nuances. For example, our findings may not have fully captured the views of nurses about working with Pakistani patients, which may have emerged with a greater number of interviews. However, the data analysis discussion group included an HCP who provided some context that enabled a more nuanced interpretation of the views expressed. For example, over-generalisation in some of the perspectives could also be seen as genuine awareness of cultural diversity, albeit, blunted by the lack of appropriate training.

Exploring the asthma care of specific subcultural groups of Bangladeshi and Pakistani patients presents greater validity, but we acknowledge that there are many South Asian groups to which our findings (including concerns about Ramadan) may apply. However, logistical constraints prevented us from fully examining the perspectives on other subcultural groups. Use of reflexive notes aided quality control by highlighting the influence of the researcher’s background, such as age, gender, ethnicity, emotional responses, and political/professional beliefs, as well as raising awareness of reactions and ideas imposed during the research process. A limitation of the study was the use of selected questions from the SL-ASIA scale to describe the level of acculturation in the sample who have varied ethnic backgrounds, for example the scale included the assessment of South Asian languages. There were no other existing scales for healthcare professionals and a prior testing of face validity of the scale revealed that the whole scale was difficult to use [31,55].

## Conclusions

Awareness of and making sense of culture was seen as an important basis for adapting self-management support for Bangladeshi and Pakistani patients living with asthma, though without any formal cultural training HCPs relied on experience and generating their own theories. Overgeneralisation or stereotyping due to limited knowledge, such as reducing culture to language spoken, may have undermined perceived positive support for self-management without professional awareness, for example involving family to support young patients. HCPs widely recognised that provision for different languages were inadequate, so that family was often used as the preferred method for translation, though consent to share patient information remained assumed. Professionals believed that cultural competence could be developed by cultural training and/or updated population-specific health information. However, how to implement and develop meaningful impact in such interventions and the healthcare service remains tentative and needs further research. Ethnic-matching of provider and patients has been recommended as the bare minimum requirement to build cultural competence [17], however this work challenges this, and asks why ethnic-matching takes priority over other demographic factors such as age, gender, technical, and interpersonal skills [56]. Perhaps, matching providers and patients from various acculturation groupings or other demographic factors, alongside ethnicity may be beneficial. Solutions will of necessity be complex and dynamic to address the nuances of cultural diversity, but our findings suggest that they will be welcomed by HCPs who seek to support people from minority populations.

## Supporting information

S1 Checklist COREQ (COnsolidated criteria for REporting Qualitative research) checklist. (PDF)

S1 Table Selected themes, subthemes, and illustrative quotes. (DOCX)
