# Supplementary material for: Exploring the perspectives of healthcare professionals on providing supported asthma self-management for Bangladeshi and Pakistani people in the UK
Source: PLoS One. 2024 Jun 10;19(6):e0302357. doi: 10.1371/journal.pone.0302357 (PMC11164332; doi:10.1371/journal.pone.0302357)
Supplement: S1 Table — (DOCX) [file pone.0302357.s002.docx]

**Supporting Information 1 Table.** **Selected themes, subthemes, and illustrative quotes**

| Themes | Subthemes | Illustrative quotes |
| --- | --- | --- |
| Theme 1. Professional insights on patients | Insights on Bangladeshi/Pakistani culture | Anxieties - *“…and were very anxious generally, and had so many anxieties” (Consultant, participant 5)* |
|  | Insights on individual as a self-manager | Illness beliefs - *“They've got to have their flu jabs. But then you get the mum and dad bringing them in but then they suddenly say, 'But it's got the gelatine in it'… but they still, but we still struggle with that” (Primary care nurse, participant 8)* |
| Theme 2. Making sense of culture | Tuning into culture | Theorisations - *“And then, there are presumably cultural factors as well. I don't know what those are, I can only guess at those, this really, I don't know, which is presumably what you're investigating. There's, I think there is lower compliance, for whatever reason, because that is the crux of, when you look at most patients with difficult asthma whatever, what, whatever ethnicity they're from, one of the biggest problems to deal with is that, patients tend to be, less compliant. And, like you've said is you know we have a bigger proportion of, Asian subcon- Indian Subcontinent patients with difficult asthma. And compliance is an issue that is my impression from what I've seen. I don't know why that is though, but they tend to be less compliant people, for whatever language and cultural reasons there are, level of understanding I don't know” (Consultant, participant 6)* |
|  | Contemplating holistic support | Accounting for various ethnicities - *“I don't know, I mean there's a language thing and that doesn't apply just to that, to that community obviously, you know I have patients from Eastern Europe and other places with, with very poor English patients who were born in Pakistan or Bangladesh or whatever. Especially who are elderly for example” (Consultant, participant 6).* |
| Theme 3. Approaches to supported self-management | Integrating perceived cultural values into support | Dealing with emotions - *“So, depression and anxiety is a big thing, I think there's a cultural barrier to accessing help, for those things, and what I see in those communities is that those individuals, do not want to have, access counselling that we offer. And even though we have a, therapist in the clinic with us to help, but they don't want to, they don't want to, access that” (Consultant, participant 6)* |
|  |  | Attitudes towards complementary and alternative medicine - *“Like the other day someone came in, they were a little elderly lady in a wheelchair, and a patient was using a bit less painkillers and I went, 'Oh what’s making her do this?' And the, other patient's, the son said, ‘She’s done cupping’, and it was, and I saw the cupping on her legs and I thought, ‘Oh my God, I know nothing about this medicine’, and she believed in it” (GP, participant 3)* |
|  | Supporting barriers to understanding | Building a relationship - *“…I'm not a psychologist but I think if they build up a relationship with you, you've broken down so many barriers, and just getting to grips with it” (Nurse specialist, participant 7)* |
| Theme 4. Challenges of supported self-management | Professional role boundaries | Level of exposure to Bangladeshi/Pakistani patients - *“I've hardly had any Pakistani patients, and I know very little about them as a group, I've only got like, I think in this practice, I think I've only got like five families, so I wouldn't be able to judge, or say anything about them” (Consultant, participant 6)* |
|  |  | Time restrictions - *“We don't really get time in the consultation to, do that as the doctor, we're very much more focused on the medication, if we're lucky enough to have a nurse specialist with us in clinic, and that's ideal to have one in every asthma clinic so that at least even if you're, if it's only for your new appointment, that they have more time to really go into the, person's daily life, and you know all of those things” (Consultant, participant 5)* |
|  |  | Working outside the system - *“I think I go in possibly too much. Instead of just drawing a line, because in a 15-minute consultation in clinics I'm thinking, we've only got 15 minutes, which ends up being 30 minutes, like girl last week, knew more she said. She told me a lot more than her family and best friends. But she learned a lot, going away, because we've explored about smoking, guys, lifestyle changes, compliance” (Nurse specialist, participant 7)* |
|  | Service restrictions | Working with other professionals - *“I think because we've got a highly specialised within the hospital, there isn't a, there isn't a lot of, joint working” (Consultant, participant 5)* |
|  |  | Access and patient pathways - *“We’re expected in the primary care to, manage asthma very well, am I allowed to refer people? Then say, ‘Am I allowed to refer people? Why aren’t you looking after this?’ But sometimes diagnosis can be difficult” (GP, participant 1)* |
